# Bidirectional associations between adiposity and physical activity: a longitudinal study from pre-puberty to early adulthood

**DOI:** 10.3389/fendo.2023.1135852

**Published:** 2023-06-19

**Authors:** Shenglong Le, Timo Törmäkangas, Xiuqiang Wang, Si Man Lei, Niels Christian Møller, Jan Christian Brønd, Niels Wedderkopp, Petri Wiklund, Sulin Cheng

**Affiliations:** ^1^ Exercise Health and Technology Center, Physical Education Department, Shanghai Jiao Tong University, Shanghai, China; ^2^ Department of Physical Therapy, Taihe Hospital, Hubei University of Medicine, Shiyan, China; ^3^ Exercise Translational Medicine Center, Shanghai Center for Systems Biomedicine, Shanghai Jiao Tong University, Shanghai, China; ^4^ Faculty of Sport and Health Sciences, University of Jyväskylä, Jyväskylä, Finland; ^5^ Faculty of Education, University of Macao, Macao, China; ^6^ Centre of Research in Childhood Health, Research Unit for Exercise Epidemiology, Department of Sports Science and Clinical Biomechanics, University of Southern Denmark, Odense, Denmark; ^7^ The Research Unit of Pediatrics, Department of Clinical Research, University of Southern Denmark, Odense, Denmark; ^8^ Huawei Helsinki Research Center, Huawei Technologies Oy (Finland) Co. Ltd, Helsinki, Finland

**Keywords:** adiposity, body composition, puberty, physical activity, longitudinal study

## Abstract

**Objective:**

This study aimed to investigate directional influences in the association between adiposity and physical activity (PA) from pre-puberty to early adulthood.

**Methods:**

In the Calex-study, height, weight, body fat and leisure-time physical activity (LTPA) were measured at age11.2-years, 13.2-years and 18.3-years in 396 Finnish girls. Body fat was measured by dual-energy X-ray absorptiometry, calculating fat mass index (FMI) as total fat mass in kilograms divided by height in meters squared. LTPA level was evaluated using a physical activity questionnaire. In the European Youth Heart Study (EYHS), height, weight and habitual PA were measured at age 9.6-years, 15.7-years and 21.8-years in 399 Danish boys and girls. Habitual PA and sedentary behaviour were assessed with an accelerometer. Directional influences of adiposity and PA were examined using a bivariate cross-lagged path panel model.

**Results:**

The temporal stability of BMI from pre-puberty to early adulthood was higher than the temporal stability of PA or physical inactivity over the same time period both in girls and boys. In the Calex-study, BMI and FMI at age 11.2-years were both directly associated with LTPA at age 13.2-years (β = 0.167, p = 0.005 and β = 0.167, p = 0.005, respectively), whereas FMI at age 13.2-years showed an inverse association with LTPA at age 18.3-years (β = - 0.187, p = 0.048). However, earlier LTPA level was not associated with subsequent BMI or FMI. In the EYHS, no directional association was found for physical inactivity, light-, moderate-, and vigorous-PA with BMI during the follow-up in girls. In boys, BMI at age 15.7-years was directly associated with moderate PA (β = 0.301, p = 0.017) at age 21.8-years, while vigorous PA at age 15.7-years showed inverse associations with BMI at age 21.8-years (β = - 0.185, p = 0.023).

**Conclusion:**

Our study indicates that previous fatness level is a much stronger predictor of future fatness than level of leisure-time or habitual physical activity during adolescence. The directional associations between adiposity and physical activity are not clear during adolescence, and may differ between boys and girls depending on pubertal status.

## 1 Introduction

It is widely believed that secular declines in physical activity (PA) level have greatly contributed to the increased prevalence of overweight and obesity in children and adolescents (1–4). However, studies of the association between PA and body composition in these populations have shown inconsistent results (2–4), which may be caused by methodological issues, including cross sectional designs with low power, and imprecise measurements of PA and body composition as well as idiosyncratic factors limit participation of PA. It is well known that cross-sectional studies are limited in their ability to detect directional influences of variables on each other, and the inverse association between PA and adiposity could also be explained by reverse causation where increased adiposity causes reduced PA rather than vice versa. Furthermore, body composition has been shown to track from childhood to adulthood; a small number of longitudinal studies have found that adiposity predicted decreased PA or increased physical inactivity indicating the potential reverse direction of causality between adiposity and activity in pre-pubertal children (5–7), but only one of them has showed bidirectional associations between PA and adiposity in adolescents, with a short follow-up duration from early puberty to mid-puberty (7).

The cross-lagged panel model is a useful and accepted tool for examining the directional relations of repeated measurements in longitudinal panel data (8). However, only a few studies have used this model to investigate associations between physical activity and fatness, and most of them have concerned adults (9, 10) or elderly patients (8). Two studies that did look at children and adolescents examined the relationship between engagement in PA and the development of motor competence (11) and associations between perceived parental support and moderate to vigorous PA (12), but neither study considered relationships with adiposity. Hence further evidence is needed to verify the relationship between adiposity and PA in order to find the right strategy to tackle these two issues.

In the present study, we aimed to investigate bidirectional associations between adiposity and PA in girls and boys during their transition from childhood to early adulthood using cross-lagged path model analysis.

## 2 Methods and materials

### 2.1 Study design and participants

This study includes two longitudinal cohorts. The Calex-study is a longitudinal family study aiming to understand how bone, fat, and muscle develop from childhood into adulthood. The study design and participant recruitment are described in detail in our earlier reports (13, 14). In brief, a total of 396 girls participated in this study from the city of Jyväskylä and Central Finland for an average duration of 7.5 years. Data collection was performed at the age of 11.2 years (baseline), and 13.2 years (n=202) and 18.2 years (n=236) at follow-ups. All measurements were performed within a 2- Exclusion criteria when children were sampled at b week period in the same month at each time to avoid seasonal effects. The study protocol was approved by the ethical committees of the Central Hospital of Central Finland (memo 22/8/2008). The study was conducted in accordance with the declaration of Helsinki. Written informed consent was obtained from all participants and their parents or legal guardians before the study.

The other cohort is the European Youth Heart Study (EYHS), which is an international, population-based, multicentre study that addresses cardiovascular disease risk factors in children and adolescents. A detailed description of the EYHS has been published elsewhere (15). Study participants were initially enrolled at age 9 years (3rd grade). And follow-ups were conducted at 15- and 21 years of age. The eligible cohort for the current analyses was n=133 individuals including both girls (n=77) and boys (n=56) who had complete data on all exposure and outcome variables. At the baseline (3rd graders), if children were not enrolled in a regular class in Danish primary municipality schools (i.e., children from special classes with specific physical- or psychological challenges or chronical illnesses, as well as children attending private schools) there were excluded from this study. The study was carried out in accordance with The Helsinki Declaration and approved by the Regional Scientific Ethical Committees for Southern Denmark (EYHS-I: case no. 96/272, EYHS-II: S-VF-20030067, EYHS-III: S-20090100).

### 2.2 Anthropometric and body composition assessment

In the Calex-study, all measurements were performed after overnight fasting. Body weight was measured using an electronic scale, and height using a stadiometer with an accuracy of 0.1 kg and 0.1 cm, respectively. BMI was calculated. In addition, dual-energy X-ray absorptiometry (DXA) (Prodigy, GE Lunar Corp., Madison. WI, USA with software version 9.3) was used to measure whole body fat mass. Precision of the repeated measurements of DXA expressed as coefficient of variation was 2.2% for fat mass. Fat mass index (FMI) was calculated as total fat mass in kilograms divided by height in meters squared.

In the EYHS, height and weight were measured to the nearest 0.1 cm and 0.1 kg, respectively using standard anthropometric procedures (SV-Seca 710 stadiometer and sliding weight, Seca Precision for Health, Hamburg Germany), with the participants wearing underwear and no shoes. BMI was calculated.

### 2.3 Physical activity assessment

In the Calex-study, leisure-time physical activity (LTPA) level was evaluated using a modified version of a World Health Organization (WHO)-validated self-administrated physical activity questionnaire described in our earlier study (16). The modifications in the questionnaire included questions about frequency and duration of PA: specifically, the first, second and third favourite physical activities that subjects participated outside of school, the duration per exercise session, and times per week. This could include both sports and other more informal physical activities. The intensity of each activity was calculated on the basis of the energy expenditure per minute. An LTPA score was calculated summing over the product of activity frequency (times per week) times intensity index (metabolic equivalents) times loading of the three physical activities (16). The LTPA scores were validated against activity energy expenditure estimated from doubly-labelled water and indirect calorimetry (n = 17, r = 0.651) at the 7.5-year follow-up. Physical inactivity was obtained from questionnaire and calculated as the sum of sitting and lying hours per day.

In the EYHS study, total habitual PA and sedentary behaviour was assessed with an accelerometer (EYHS-I & II: ActiGraph, model 7164; ActiGraph, Walton Beach, FL, EYHS-III: Actigraph, model GT1M or GT3X, Pensacola, Florida, USA). The accelerometer was worn on the right hip with an elastic band during the whole day (except sleeping and water-activities) for five consecutive days. Custom-written Propero software was used to process the raw data of accelerometer, scanning the data files for abnormal values and periods with zero activity to generate an individual average PA value (counts/min) (17). Non-wearing of the device was identified by at least 45 consecutive minutes of zeroes. Children and adolescents who did not accumulate at least 600 registered min/day for at least 3 days were excluded from further analysis. The accelerometer method has been validated in both children and adolescents against various criteria and found to be a valid method (r = 0.54–0.66) for assessment of habitual PA in epidemiological studies (18, 19). However, both studies are with limited sample size and did not take into account gender differences. The light- (LPA), moderate- (MPA), and vigorous-PA (VPA) cut-off windows were set at 100-1999, 2000-3999, 4000-50000 counts per minute, respectively, using 60 second epochs. The time below the 100 counts per minute light-PA cut-off point was used to quantify the subject’s sedentary behavior. The PA values in EYHS study reflect total habitual PA which included LTPA and other types of PA.

### 2.4 Statistical procedures

Normality of the data was checked by histograms and Q-Q probability plots to assess if the data violated the assumption of normality using IBM SPSS Statistics for Windows, Version 22.0 (IBM Corp, Armonk, NY, USA). If the variables were not normally distributed, their natural logarithms were used. Means and standard deviations were calculated for continuous variables that approximated a normal distribution. Analysis of variance (ANOVA) was used to analyse differences in adiposity among groups of different levels of physical activity. The directional influence of the associations was examined using a bivariate cross-lagged path panel model, in which the auto-regressive part of the model indicates the temporal stability of the variables from one time point to the next, and cross-lagged paths are used to assess reciprocal relationships between the variables at consecutive time points (7, 20). In addition, discrete-time structural equation modelling was used to examine the cross-lagged paths between physical activity and adiposity over time, while controlling for cross-time stability of each of the variables. Model fitting was conducted with Mplus version 7.17. The model fit was assessed by the following statistics: the χ2 statistic (p > 0.05 for good fit), the comparative fit index (CFI, > 0.95 for good, > 0.90 for adequate), and standardized root mean square residual (SRMR, < 0.08 for good) (21, 22).

## 3 Results

General characteristics of the Calex-study and the EYHS participants are shown in **Tables 1**, **2**. In the Calex-study, body weight, height, BMI and FMI increased significantly from childhood to early adulthood (all p < 0.001). The average LTPA score and the average time spent in sedentary activities did not change significantly during the follow-ups. In the EYHS study, LPA, MPA and VPA decreased, and physical inactivity increased, from the age of 9.5 years to the age of 21.7 years (all p < 0.001, except for VPA, p < 0.01). Compared to girls, boys spent more time in MPA at each time point and in VPA at the age of 9.7 years and 15.7 years (all p < 0.05). The trajectories of the individual participants are shown in the **Supplementary Figures 1–3**.

**Table 1:** General characteristics of study participants in the Calex-study (mean ± SD).

	Baseline (n = 258)	2-Year FU (n = 222)	7-Year FU (n =236)
Age (years)	11.2 ± 0.75	13.2 ± 0.75	18.3 ± 1.08***
Height (cm)	145.6 ± 8.0	157.9 ± 7.0	165.8 ± 5.7***
Weight (kg)	39.19 ± 8.66	49.98 ± 10.52	60.29 ± 9.97***
BMI (kg/m^2^)	18.32 ± 2.91	19.96 ± 3.49	21.88 ± 3.15***
Fat mass (kg)	10.70 ± 5.64	13.81 ± 7.32	19.28 ± 7.42***
FMI (kg/m^2^)	4.8 ± 2.4	6.0 (3.2)	7.0 ± 2.6***
LTPA (hrs/day)	2.8 ± 2.1	3.1 ± 2.0	3.6 ± 2.2
LTPA score	93.5 ± 88.8	104.8 ± 89.5	90.1 ± 88.2
PIA (hrs/day)	18.2 ± 1.9	18.3 ± 2.3	17.7 ± 2.9

SD, standard deviation; FU, follow-up; BMI, body mass index; FMI, fat mass index; LTPA, leisure-time physical activity; PIA, physical inactivity.

***, compared with the baseline, p<0.001.

**Table 2:** General characteristics of study participants in the EYHS study (mean ± SD).

	Girls			Boys		
	Baseline (n = 77)	6-Year FU (n = 77)	12-Year FU (n = 77)	Baseline (n = 56)	6-Year FU (n = 56)	12-Year FU (n = 56)
Age (years)	9.5 ± 0.36	15.6 ± 0.31	21.7 ± 0.36	9.7 ± 0.35	15.7 ± 0.34	21.8 ± 0.32
Height (cm)	138.4 ± 6.5	166.0 ± 7.1	167.6 ± 7.3**	141.1 ± 5.7	177.7 ± 7.2	181.7 ± 6.1**
Weight (kg)	33.0 ± 5.2	57.9 ± 8.5	66.5 ± 10.9**	33.9 ± 4. 8	65.9 ± 9.4	79.3 ± 12.9**
BMI (kg/m^2^)	17.1 ± 2.0	21.0 ± 2.3	23.7 ± 3.2**	17.0 ± 1.7	20.9 ± 2.6	24.0 ± 3.6**
LPA (hrs/day)	6.40 ± 1.49	5.14 ± 1.14	4.45 ± 1.29**	6.31 ± 1.45	5.10 ± 1.33	4.24 ± 1.55**
MPA (hrs/day)	0.66 ± 0.46	0.48 ± 0.28	0.36 ± 0.24**	0.88 ± 0.39	0.65 ± 0.29	0.46 ± 0.25**
VPA (hrs/day)	0.22 ± 0.25	0.18 ± 0.14	0.16 ± 0.18	0.32 ± 0.34	0.29 ± 0.27	0.19 ± 0.17*
PIA (hrs/day)	5.91 ± 1.92	8.23 ± 1.19	8.81 ± 1.43**	5.96 ± 2.11	8.38 ± 1.54	8.96 ± 1.61**

SD, standard deviation; FU, follow-up; BMI, body mass index; LPA, light physical activity; MPA, moderate physical activity; VPA, vigorous physical activity; PIA, physical inactivity.

*, compared with the baseline, p<0.01.

**, compared with the baseline, p<0.001.

**Figure 1:**
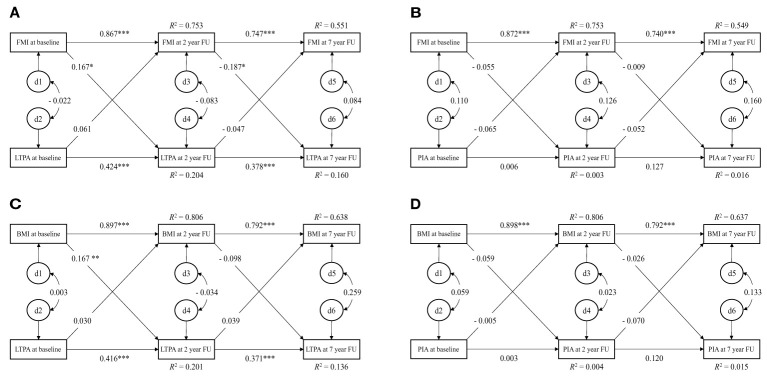
Cross-lagged path model for leisure-time physical activity and FMI and BMI in the Calex-study. d = residual variance. **(A)**: χ2 = 2.709, p = 0.608, CFI = 1.000, SRMR = 0.032; **(B)**: χ2 = 1.050, p = 0.902, CFI = 1.000, SRMR = 0.020; **(C)**: χ2 = 5.787, p = 0.216, CFI = 0.997, SRMR = 0.038; **(D)**: χ2 = 4.468, p = 0.346, CFI = 0.999, SRMR = 0.028. FMI, fat mass index; LTPA, leisure-time physical activity; PIA, physical inactivity; BMI, body mass index; CFI, comparative fit index; SRMR, standardized root mean square residual. Note: Discrete-time structural equation modelling was used to examine the cross-lagged paths between physical activity and FMI and BMI over time, while controlling for cross-time stability of each of the variables. The model fit was assessed by the following statistics: the χ2 statistic (p > 0.05 for good fit), CFI (> 0.95 for good, > 0.90 for adequate), and SRMR (< 0.08 for good). * p < 0.05; ** p < 0.01; *** p < 0.001.

In the Calex-study, we found that FMI/BMI strongly predicted subsequent BMI/FMI at each measurement wave (model R-square ranged from 0.551 to 0.806, p < 0.001 for all) (**Figure 1**). A similar pattern was observed for LTPA (**Figures 1A, C**) although with notably smaller R-square (R2 = 0.136 - 0.204, p < 0.001 for all), but not for physical inactivity (p > 0.05 for all) (**Figures 1B, D**). This autoregressive part of the model indicates that the temporal stability of BMI from childhood to early adulthood is higher than the temporal stability of LTPA or physical inactivity over the same period.

In the Calex-study, the cross-lagged associations showed that higher FMI at the age of 11.2 years predicted higher LTPA at the age of 13.2 years (β = 0.167, p = 0.005), but higher FMI at the age of 13.2 years predicted lower LTPA at the age of 18.3 years (β = - 0.187, p = 0.048). LTPA did not predict subsequent FMI at any time point (**Figure 1A**). Similar to FMI, higher BMI at the age of 11.2 years predicted higher LTPA at the age of 13.2 years (β = 0.167, p = 0.005) (**Figure 1**), but LTPA did not predict subsequent BMI (**Figure 1C**). Both models had good fit to the data (FMI: χ2 = 2.709, p = 0.608; CFI = 1.000; RMSEA = 0.032; BMI: χ2 = 5.787, p = 0.216; CFI = 0.997; SRMR = 0.038). No directional influence was found between physical inactivity and FMI/BMI during the follow-up (**Figures 1B, D**).

**Figure 2:**
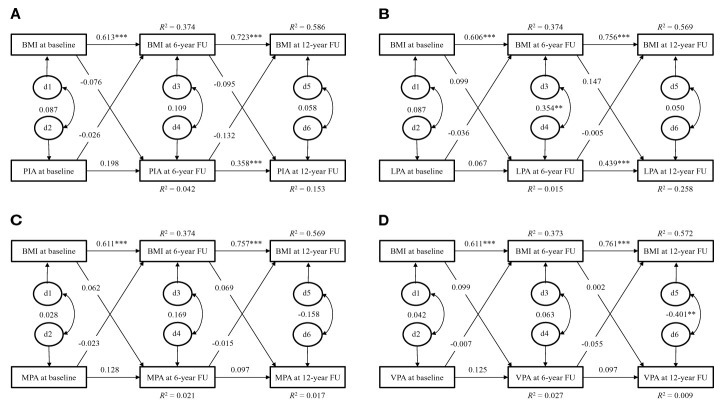
Cross-lagged path model for physical activity and BMI of girls in the EYHS. d = residual variance. **(A)**: χ2 = 10.756, p = 0.029, CFI = 0.945, SRMR = 0.048; **(B)**: χ2 = 9.497, p = 0.050, CFI = 0.958, SRMR = 0.047; **(C)**: χ2 = 13.343, p = 0.010, CFI = 0.913, SRMR = 0.055; **(D)**: χ2 = 7.258, p = 0.123, CFI = 0.971, SRMR = 0.040. BMI, body mass index; PIA, physical inactivity; LPA, light physical activity; MPA, moderate physical activity; VPA, vigorous physical activity; CFI, comparative fit index; SRMR, standardized root mean square residual. Note: Discrete-time structural equation modelling was used to examine the cross-lagged paths between physical activity and BMI over time, while controlling for cross-time stability of each of the variables. The model fit was assessed by the following statistics: the χ2 statistic (p > 0.05 for good fit), CFI (> 0.95 for good, > 0.90 for adequate), and SRMR (< 0.08 for good). ** p < 0.01; *** p < 0.001.

**Figure 3:**
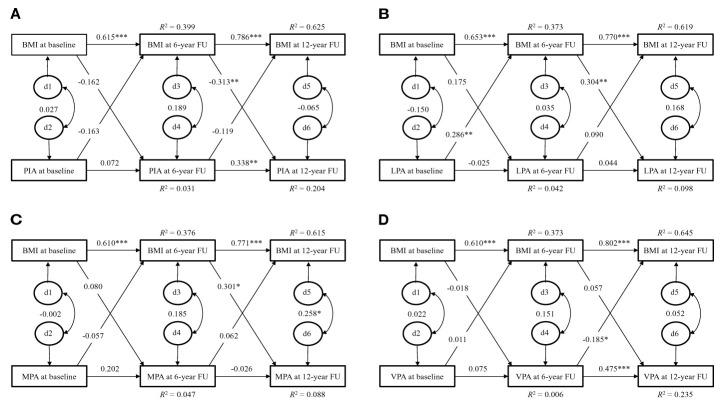
Cross-lagged path model for physical activity and BMI of boys in the EYHS. d = residual variance. **(A)**: χ2 = 14.909, p = 0.005, CFI = 0.892, SRMR = 0.061; **(B)**: χ2 = 13.775, p = 0.008, CFI = 0.898, SRMR = 0.055; **(C)**: χ2 = 5.198, p = 0.268, CFI = 0.986, SRMR = 0.043; **(D)**: χ2 = 5.310, p = 0.257, CFI = 0.985, SRMR = 0.037. BMI, body mass index; PIA, physical inactivity; LPA, light physical activity; MPA, moderate physical activity; VPA, vigorous physical activity; CFI, comparative fit index; SRMR, standardized root mean square residual. Note: Discrete-time structural equation modelling was used to examine the cross-lagged paths between physical activity and BMI over time, while controlling for cross-time stability of each of the variables. The model fit was assessed by the following statistics: the χ2 statistic (p > 0.05 for good fit), CFI (> 0.95 for good, > 0.90 for adequate), and SRMR (< 0.08 for good). * p < 0.05; ** p < 0.01; *** p < 0.001.

In the EYHS, the temporal stability of BMI (β = 0.606 - 0.802, p < 0.001 for all) from childhood to early adulthood was also higher than the temporal stability of total habitual PA which included LTPA (β = - 0.025 - 0.475, p > 0.05 for all, except p < 0.001 for LPA of girls and VPA of boys from age 15 to 21) or physical inactivity (β = 0.072 - 0.358, p > 0.05 for all, except p < 0.001 for girls from age 15 to 21 and p = 0.003 for boys from age 15 to 21) over the same time period in both girls and boys (**Figures 2**, **3**).

In the girls of EYHS, no directional influence was found between physical inactivity, LPA, MPA, and VPA and BMI during the follow-up (**Figure 2**).

In the boys of EYHS, the cross-lagged associations showed that higher BMI at the age of 15.7 years predicted less physical inactivity at the age of 21.8 years (β = - 0.313, p = 0.007) with good model fit (χ2 = 14.909, p = 0.005, SRMR = 0.061, **Figure 3A**). Greater LPA at the age of 9.7 years predicted higher BMI at the age of 15.7 years (β = 0.286, p = 0.004) and higher BMI at the age of 15.7 years predicted more LPA at the age of 21.8 years (β = 0.304, p = 0.014) with good model fit (χ2 = 13.775, p = 0.008, SRMR = 0.055, **Figure 3B**). In addition, higher BMI at the age of 15.7 years predicted greater MPA (β = 0.301, p = 0.017) at the age of 21.8 years with good model fit (χ2 = 5.198, p = 0.268, SRMR = 0.043, **Figure 3C**). However, higher VPA at the age of 15.7 years predicted lower BMI at the age of 21.8 years (β = -0.185, p = 0.023) with good model fit (χ2 = 5.310, p = 0.257, SRMR = 0.037, **Figure 3D**). No other directional associations were found between physical inactivity, MPA and VPA and BMI during the follow-up.

## 4 Discussion

In the present study, we investigated bidirectional cross-sectional and prospective associations between adiposity and physical activity in girls and boys followed from childhood to early adulthood. We found no clear bidirectional associations, but our results suggested a low-to-modest association of adiposity with future physical activity level in girls of the Calex-study, and BMI modestly associated with future light and moderate physical activity in boys of the EYHS study. No statistically significant effects were found in girls of the EYHS study. No inactivity predicted the level of future adiposity. To our knowledge, this is the first study to suggest directional associations between fatness and physical activity in girls and boys transitioning through puberty.

The transition from childhood to early adulthood is a period characterized by marked changes in body composition and physical activity (23, 24); in early adolescence muscle mass increases significantly among boys and fat deposition increases among girls (23), while physical activity declines in both sexes, more so in boys than girls (24, 25) despite the fact that boys still engage in more PA than girls in absolute terms. Physical activity levels in adolescence have been found to be inversely associated with fatness in most longitudinal studies, and this has been interpreted as evidence of a protective effect of physical activity against excess body fat accumulation in youth (26, 27). This was to some extent supported by our results in boys where more time spent in VPA at the age of 15.7 years was found to predict lower BMI at the age of 21.8 years. However, our prospective analyses also provide some evidence for the inverse relationship between physical activity and fatness during pubertal growth suggesting that the relationship may be dominated by the effect of adiposity on future physical activity rather than the effect of physical activity on future adiposity, especially for girls. In the Calex-study, the influence of FMI on LTPA was statistically significant from pre-puberty to early adulthood for girls, although the direction of the association (sign) changed, whereas the effect of BMI on LTPA was inconsistent, showing significant associations only at baseline. In addition, PA assessments in the EYHS study, which were not restricted only to leisure time activity, also showed the temporal rank-order was more stable for BMI from childhood to early adulthood was higher than the temporal stability of total habitual PA over the same time period in both girls and boys. The inconsistent associations may be explained by the fact BMI incorporates both fat mass and lean mass, which both increase during pubertal growth, and are likely to be associated with physical activity in opposite directions as well as large variations in LTPA.

Our findings are to some degree in agreement with earlier prospective studies that examined bidirectional associations between adiposity and physical activity which showed that adiposity predicted physical activity or physical inactivity in pre-pubertal children (5, 6, 28) and in adults (29, 30). However, one study has addressed the “bi-directional hypothesis” in adolescents, and in that study, Halal and colleagues found no statistically significant directional association between adiposity and physical activity measured at 11 and at 14 years of age in a large number of Brazilian boys and girls (7). Our data seems to be partly in line with this study. In our Calex-study, we found that adiposity predicted future LTPA, whereas in the EYHS the findings are ambiguous with no clear directional association. The reason for the discrepancies between our results and those of Halal et al. is not clear, but it could be due to differences in follow-up durations, body composition and PA assessment methods. It is also possible that when girls transit from pre-puberty to post puberty, their body composition rapidly develops in all three compartments which may override the influence of PA (13). Furthermore, physical activity behaviours may also differ between high and middle-income countries, which may lead to variations in associations between physical activity and body composition observed across different countries (such as high and middle-income) during adolescence (31). In contrast to our findings, Riddoch and colleagues found that physical activity level at 12 years correlated inversely with fat mass at 14 years in boys and girls, but they did not test the reverse association and therefore were unable to determine the dominant direction of association (32). In the present study, BMI and body composition tracked more strongly over time than either physical activity or inactivity, which was expected as the transition from adolescence to adulthood is characterised by considerable physical and psychosocial changes and participation in physical activity is a matter of choice whereas tracking of BMI and body composition reflect in part the genotype and the long-term impact of early life exposures. These results are in agreement with the few other studies which have examined tracking of both body composition and physical activity in this age group (7, 33), and suggest that LTPA may have limited capacity to modify body composition during pubertal development (16). Hence in addition to increasing PA, attention should be also paid to factors such as socioeconomic status and excess energy intake (34). Regarding the prevention of adolescent overweight, in the Calex-study, the prevalence of overweight, defined by BMI and fat% combined, was 14.8% (35) and in the EYHS, with overweight defined by using BMI alone, the prevalence was 26.0% for girls and 37.5% for boys at the age of 21.7- and 21.8-years, respectively. The WHO recommends that adolescents should accumulate at least 60 minutes per day in moderate-to-vigorous physical activity (36). This might be hard to achieve, particularly for girls, whose physical activity decreased dramatically during puberty. In the EYHS, the mean duration of MPA and VPA in girls is only 0.48 hour per day at the age of 15.6-years and 0.36 hour per day at the age of 21.7-years. Previous studies showed that only two-thirds of boys and one-quarter of girls report doing 20 min of sustained moderate-to-vigorous physical activity three times per week (37).

Even though adiposity could predict future physical activity level in both genders, our study showed that gender differences were visible in the directional association between adiposity and physical activity. In girls, more adiposity seems to predict less physical activity in future from puberty to early-adulthood, but in boys more adiposity means more physical activity and less sedentary behaviour in future. We do not know why there is a different pattern between genders in this directional association, but psychological and physiological explanations appear to be plausible. Previous studies showed that adolescent girls with lower perceived body image were unlikely to engage in physical activity (38, 39). It is possible that overweight girls perceive their body image negatively and, as a result, choose not to participate in sports and exercise. Girls report greater body image dissatisfaction than their male counterparts, and, in girls, body image dissatisfaction increases in later adolescence (40, 41). For girls, lack of skills is the strongest predictor of low physical exercise, while for boys it is lack of time (42). For overweight girls, it is more difficult to master exercise and sport skills, resulting in less physical activity. Another reason may be gender differences in the coping strategies for overweight. Dieting is more frequent among women than among men, the latter tending to practice physical exercise rather than dietary changes to alter the look of their bodies and to lose weight (43, 44). Rather than looking to be thinner, some of the boys want to be larger and more muscular, which only can be achieved by exercise and sports. In addition, the methodology of assessing PA may play some role in the gender differences. For example, a questionnaire-based PA assessment is less accurate than device-based measurements. The tracking in the questionnaire-based PA assessment in the Calex-study may be partially due to memory and response strategy issues in addition to actual activity levels.

Our study has both strengths and limitations. The strength of our study is the longitudinal design in which a large number of children were followed from pre-puberty to early adulthood by use of multiple measurement waves. In the Calex-study, the seasonal variation in the results was minimized by performing data collection within a 2-week period in the same month at each assessment wave, whilst the EYHS study data were collected over a school year. In addition, we used discrete-time structural equation modelling, which allowed us to examine the cross-lagged paths between physical activity and adiposity over time, while controlling for cross-time stability of each of the variables. Limitations include assessment of body composition and physical activity with different techniques in the two studies included in this current work; in the Calex-study, the self-reported assessment of physical activity and time spent in sedentary activities did not capture some aspects of activity (notably total activity and moderate-vigorous physical activity). Self-reported physical activity may also lack the natural variation in participation of physical activity due to recall bias (45), and this may be an additional explanation for errors in tracking over measurement times and thus the potential contribution of habitual physical activity to adiposity may not have been fully reflected in the results. In the EYHS study, the PA was not restricted to leisure time activity only and was measured directly with an accelerometer. However, a limitation was that body composition was not measured by DXA and the use of accelerometers depends on the assumption that less than a week of measured physical activity was representative of the subjects’ habitual physical activity. In addition, the accelerometers used at baseline were the original version of the ActiGraph which, compared to the new version of the device, had limitations on epoch, a different way of measuring the vertical accelerations, and lower precision (46). This could have adversely influenced the results observed in the present study. Moreover, irrespectively of studies (Calex-study vs. EYHS), PA and body composition was measured with different levels of precision. Body composition measured by either BMI or DXA is more precisely registered compared to PA assessed by use of questionnaire or accelerometers. This means that the true effect of PA on body composition was biased towards the null since high measurement error on the exposure variable will cause such a bias. The problem of poor reliability in the assessment PA is already gained acknowledgement (47). Hence care is needed when trying to assess the level and direction of relationships between PA and body composition and vice versa and future study may consider having a more stable variable that also reflects accumulation of physical activity, e.g., maximal aerobic capacity (VO2max).

## 5 Conclusion

In conclusion, the temporal stability of adiposity appears to be much stronger than that of physical activity from pre-puberty to early adulthood. Adiposity may predict later PA level in girls and boys transitioning from pre-puberty to early adulthood, but the results showed no evidence of a bidirectional association between adiposity and physical (in)activity. In girls, the positive or absent association between adiposity and PA before menarche and the negative association after menarche suggests that guidance promoting physical activity to maintain a healthy body weight in children and adolescents may not fully reflect the complexity of the underlying relationships nor the importance of gender differences.

## Data availability statement

The original contributions presented in the study are included in the article/**Supplementary Material**. Further inquiries can be directed to the corresponding authors.

## Ethics statement

The studies involving human participants were reviewed and approved by the ethical committees of the Central Hospital of Central Finland (memo 22/8/2008); the Regional Scientific Ethical Committees for Southern Denmark (EYHS-I: case no. 96/272, EYHS-II: S-VF-20030067, EYHS-III: S-20090100). Written informed consent to participate in this study was provided by the participants’ legal guardian/next of kin.

## Author contributions

SL and PW were responsible for the design of the study, performed data processing, performed and interpreted the statistical analyses, and drafted the manuscript. TT, XW and SL contributed to the performing and interpretations of the statistical analyses and writing of the manuscript. NM, JB and NW collected the data of EYHS, assisted in data interpretation, and critically reviewed the manuscript. SC was responsible for the design of the Calex-study and supervised the data collection, processing and analysing procedures. She also assisted in interpretation of the data, supervised the manuscript process and finalized the manuscript. All authors approved the final manuscript.
