# Supplementary material for: Bidirectional associations between adiposity and physical activity: a longitudinal study from pre-puberty to early adulthood
Source: Front Endocrinol (Lausanne). 2023 Jun 19;14:1135852. doi: 10.3389/fendo.2023.1135852 (PMC10315841; doi:10.3389/fendo.2023.1135852)
Supplement: Supplementary file 1 [file Image_1.pdf]

Supplementary Material

Bidirectional associations between adiposity and physical activity: A longitudinal study from pre-puberty to early adulthood

Shenglong Le, Timo Törmäkangas, Xiuqiang Wang, Si Man Lei, Niels Christian Møller, Jan Christian Brønd, Niels Wedderkopp, Petri Wiklund*, Sulin Cheng*

*** Correspondence:** Petri Wiklund: petri.wiklund@huawei.com; Sulin Cheng: shulin.cheng@jyu.fi

1 Supplementary Figures

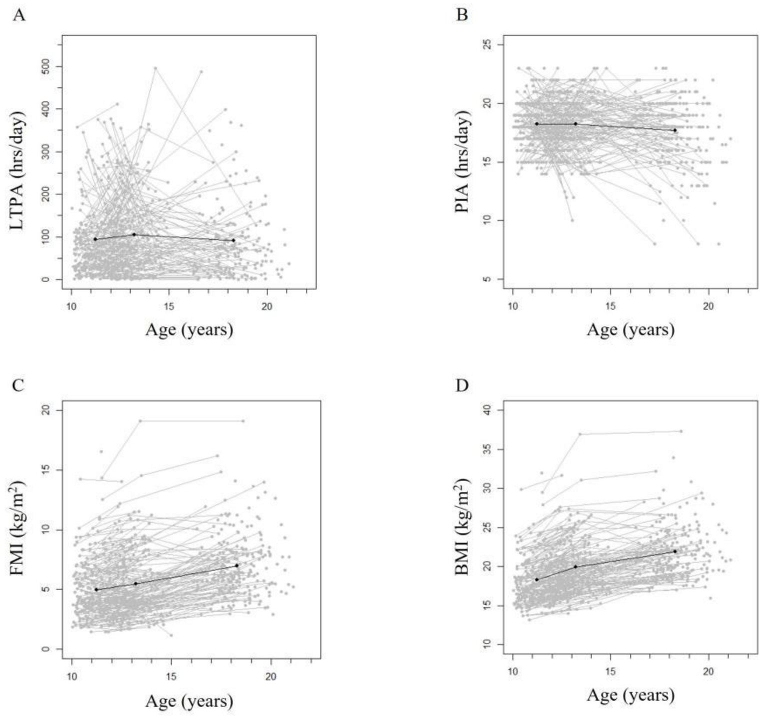

Supplementary Figure 1. Individual observed trajectories (gray) and mean of dependent variable at time point mean among Calex-study girls.

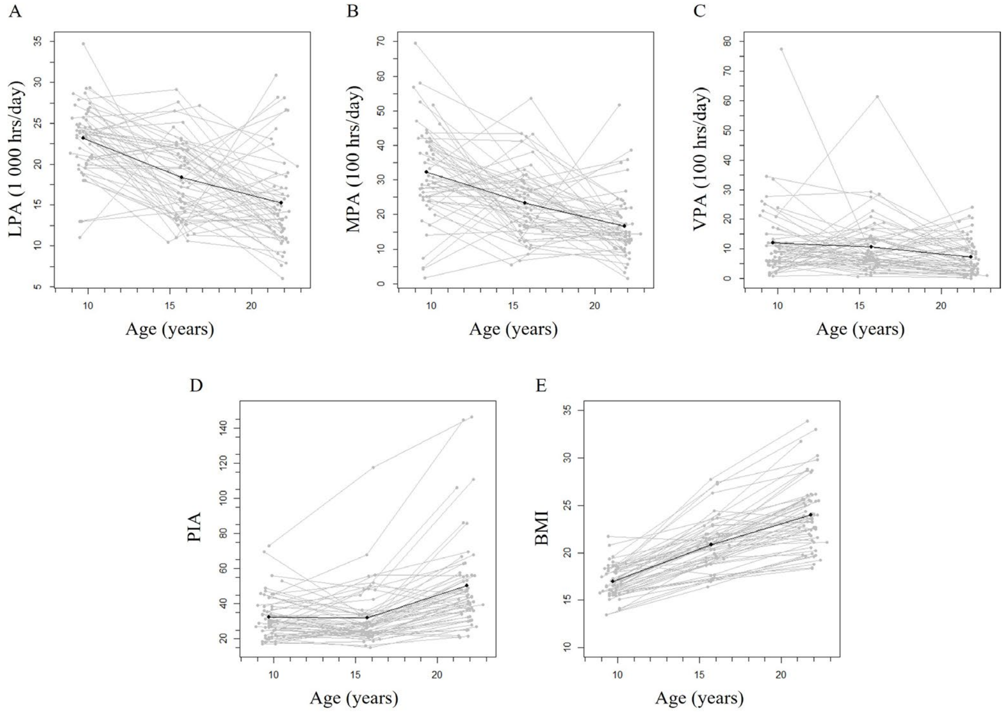

Supplementary Figure 2. Individual observed trajectories (gray) and mean of dependent variable at time point mean among EYHS boys.

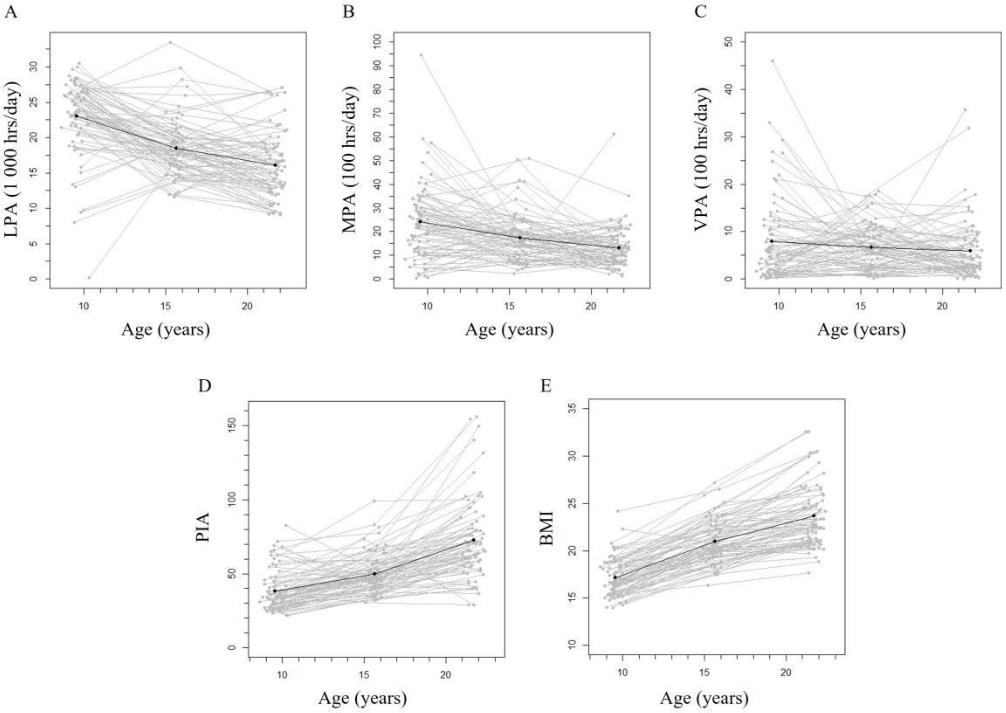

Supplementary Figure 3. Individual observed trajectories (gray) and mean of dependent variable at time point mean among EYHS girls.
